# Gastrocolic Fistula: A Rare Presentation of Colon Cancer

**DOI:** 10.1155/2018/6958925

**Published:** 2018-10-01

**Authors:** Chukwunonso Chime, Madhavi Ravi, Myrta Daniel, Harish Patel, Bhavna Balar

**Affiliations:** Division of Gastroenterology, Bronx Care Health System–Affiliate of Mount Sinai Hospital System, New York, USA

## Abstract

Gastrocolic fistulae have been described for benign conditions including penetrating peptic ulcer and complicated pancreatitis. Malignant etiology can arise from gastric or colon cancer and is a rare and late complication with an incidence of 0.3-0.4%. Usual presentation is the classic triad of weight loss, diarrhea, and feculent vomiting. Barium enema has been shown to have the highest diagnostic accuracy but endoscopy offers additional advantage of biopsy to aid in diagnosis of malignant etiology; the role of computed tomography (CT) scan is controversial. Treatment by one-stage en bloc surgical approach is the current acceptable standard of care with variable recurrence and survival rates. Adjuvant chemotherapy would be based on lymph node involvement and patient discussion.

## 1. Introduction

Gastrocolic fistula, an abnormal communication between the colon and stomach, is known in literature. Etiology varies and can be of both malignant and benign causes; they have been reported to be secondary to gastric tumors, gastric ulcers, and complication of pancreatitis [1–3]. Historically, gastrocolic fistula secondary to colon cancer is a rare and late complication [4], with incidence of malignant etiology ranging between 0.3 and 0.4% [5]. We present a case of gastrocolic fistula secondary to invasive adenocarcinoma of the transverse colon, diagnosed through an abdominal CT scan and confirmed by endoscopy with pathology. Patient was treated with en bloc resection and subsequent adjuvant chemotherapy.

## 2. Case Report

An 85-year-old woman presented to our emergency room (ER) with severe epigastric pain for one day. Pain was associated with nausea and coffee ground vomiting with feculent odor. Prior to this admission she had nonspecific abdominal discomfort with dark stools for one week and objective weight loss of 37 pounds since her last visit to the ER three years earlier. Her medical history included hypertension, diabetes mellitus, and osteoporosis. In the ER, her vitals were within normal limits, and physical examination was unremarkable except for mild abdominal tenderness and palpable prominence in the left upper quadrant. Her laboratory investigations revealed hemoglobin of 9.3g/dl, mean corpuscular volume of 76fl, white blood cell count of 9.5 k/ul, platelet count of 529 k/ul, BUN of 63 mg/dl, and creatinine of 2.1 mg/dl.

**Figure 1:**
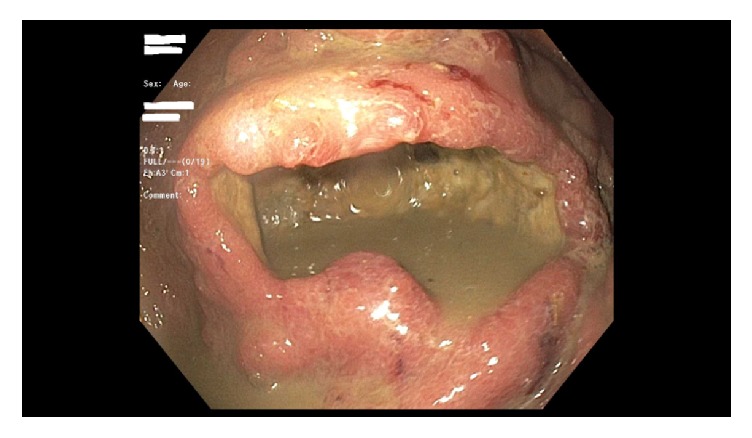
Gastric ulcer with feculent material.

**Figure 2:**
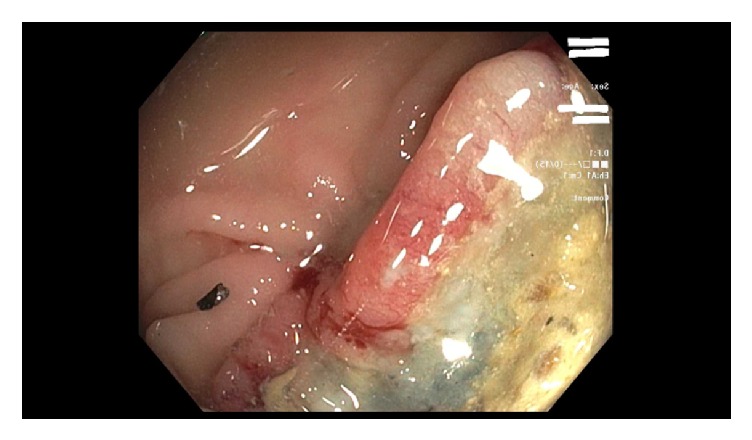
Transverse colon mass.

**Figure 3:**
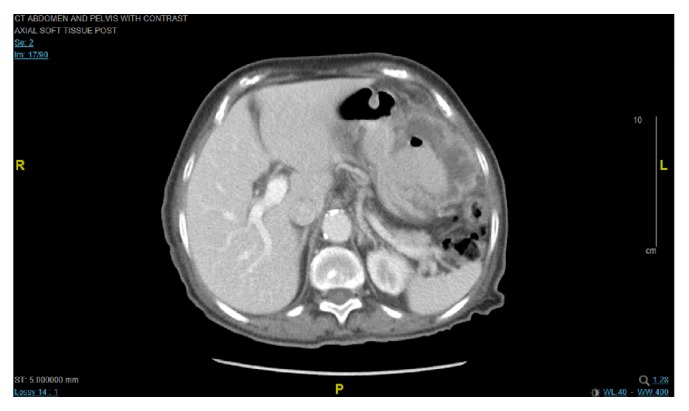
CT abdomen with IV contrast: axial image.

Computed tomography (CT) scan of the abdomen without contrast done in the emergency room showed gastric wall thickening with possible gastric mass. She was admitted to the medical service and had an upper endoscopy showing a large cratered gastric ulcer in the greater curvature of the body with excessive amount of feculent material (see Figure 1) which raised suspicion for possible fistulous connection to the large bowel. Repeat abdominal CT scan with oral and intravenous contrast confirmed suspicion of distal transverse colon mass with gastrocolic fistula (see Figure 3). Subsequent colonoscopy revealed a large, circumferential, obstructing transverse colon mass (see Figure 2). Pathology showed poorly differentiated adenocarcinoma of the colon and on immunohistochemical stain, the tumor cells were positive for CK20 and CDX2 and weakly positive for CK7, features which are consistent with colon primary. She was managed surgically with an en bloc resection of tumor with partial gastrectomy and end-to-end colonic anastomosis. Postsurgical course was uneventful and she was discharged home in stable condition. Adjuvant chemotherapy was started outpatient after risks and benefits were discussed.

## 3. Discussion

Gastrocolic fistula secondary to colon cancer is very rare; this may be attributed to prevention and early diagnosis of colon cancer with screening and surveillance colonoscopy [6]. Geographical location plays a role in etiology of malignant gastrocolic fistula, with gastric cancers being more common in Japan and eastern countries, while colon cancer is the most frequent etiology in western countries [7]. Also interestingly, sex disparity was noted when these fistulas were evaluated by geographical region; they were more common in women in western countries as compared to men in Japan and eastern countries, though age distribution was similar in the 6th and 7th decades [8]. Our index patient was a female in her 80s. The current working theories to explain this phenomenon are either the result of a direct invasion of tumor cells through the gastrocolic omentum or an inflammatory peritoneal reaction that results in adhesion and fistula formation between the two organs [9].

Our patient did not have the typical triad of diarrhea, weight loss, and feculent vomiting that characterizes gastrocolic fistula [8]. She presented with weight loss, which was described at the time as coffee ground vomiting, likely from gastrointestinal hemorrhage of the ulcer and foul eructation, which in hindsight was probably feculent material. These symptoms prompted an upper endoscopy that revealed a malignant looking gastric ulcer. Symptoms can also be nonspecific to include abdominal pain, fatigue, and nutritional deficiencies [7].

Various modalities exist for diagnosis of gastrocolic fistula, with a sensitivity of 90%; barium enema has been described as the most accurate investigative method for diagnosis [10]. Endoscopy has not fared well as a means of diagnosing these fistulas, especially small fistula that can hide between gastric folds. In a study comparing endoscopy with barium enema, consisting of 66 patients with gastrojejunocolic or gastrocolic fistulas, barium enema performed better with detection rate of 95% vs 27% for endoscopy [11]. Our patient presented with coffee ground material which prompted an upper endoscopy as initial investigation to rule out any upper gastrointestinal bleed. We believe the increased size of the fistula aided the detection of the same with endoscopy regardless of its historically low sensitivity in diagnosing this condition. The upper endoscopy also gave us an added advantage of obtaining biopsies that would help pursue the diagnosis of malignant etiology. It is unclear what role CT scan would play in diagnosis, but its added importance in preoperative planning to delineate local invasion of the primary tumor and also assess for metastatic spread cannot be overemphasized [12]. In our patient, though upper endoscopy was highly suggestive of a fistula as shown in the endoscopy images, we however proceeded with a CT scan with oral and intravenous contrast which confirmed the fistulous connection; hence there was no need for a barium enema. Subsequent colonoscopy with biopsies confirmed a transverse colon invasive adenocarcinoma of the colon as etiology of the fistula.

Treatment modalities would depend on the staging of the primary malignancy, patient's ability to withstand surgery, and the presence of metastatic spread. Surgery remains an option for patients that are good surgical candidates and has changed over time. In the 1940s, patients had two- and three-stage surgeries but this has since evolved to the current one-stage en bloc resection [13]. When colon cancer is adherent to adjacent organs, it is difficult to distinguish between malignant and inflammatory adhesions at the time of surgery, so the Practice parameters of the American Society of Colon and Rectal Surgeons recommend en bloc resection in these cases [14]. A few novel methods have been described that do not involve surgery; to mention a few, in the case of Nici et al, dual endoscopic closure was achieved with use of Resolution clips [15]. Also in a case of large inoperable malignant gastrocolic fistula and gastric outlet obstruction, Malespin et al. combined an Atrial Septal Defect closure device and a duodenal stent for palliation [16]. Though our patient's age was in the mid-80s, she was a good fit for surgical intervention and CT scan revealed clear boundaries for resection. She subsequently had an en bloc resection with good postoperative outcome and was eventually discharged home. The need for adjuvant chemotherapy would usually depend on lymph node involvement and patient's decision after discussion of benefits and risks. Our patient was started on chemotherapy after discharge as lymphovascular invasion was noted but no lymph nodes were submitted or found in the pathological specimen.

## 4. Conclusion

Invasive adenocarcinoma of the distal transverse colon due to its close proximity to the greater curvature of the stomach rarely can present with a gastrocolic fistula. Diagnosis can be made with imaging studies like barium enema or CT scan, with barium enema being the preferred approach. Endoscopic studies allow for direct visualization and opportunity to obtain biopsies. Treatment approach is usually en bloc surgical resection with favorable outcomes and variable recurrence and survival rates. Decision regarding chemotherapy should be individualized with lymph node involvement playing a crucial role.
